# Chronic diarrhea associated with persistent norovirus excretion in patients with chronic lymphocytic leukemia: report of two cases

**DOI:** 10.1186/1471-2334-11-131

**Published:** 2011-05-17

**Authors:** Todd Capizzi, Grace Makari-Judson, Richard Steingart, Wilson C Mertens

**Affiliations:** 1 Baystate Regional Cancer Program/Tufts University School of Medicine, 3400 Main Street, Springfield, MA 01107 USA

## Abstract

**Background:**

Chronic diarrhea in patients treated with immunosuppressive agents or suffering from immunosuppressive disease can represent a diagnostic and therapeutic challenge to the clinician. Norovirus infection, a major cause of acute epidemic diarrhea, has been described as a cause of chronic diarrhea in patients who are immunosuppressed, including transplant recipients and the very young.

**Case presentations:**

We describe two patients, a 64 year-old man and a 59 year-old woman, both suffering from chronic lymphocytic leukemia and hypogammaglobulinemia, who developed chronic diarrhea resistant to therapy. In both cases, after months of symptoms, persistent norovirus infection--documented by repeatedly-positive high-sensitivity stool enzyme immunoassay--was found to be the cause. Both patients died with active diarrheal symptoms.

**Conclusions:**

We describe the first cases of advanced chronic lymphocytic leukemia to suffer from chronic symptomatic norovirus infection. Clinicians caring for such patients, particularly those with concomitant hypogammaglobulinema, who have chronic unexplained diarrhea, should consider norovirus infection in the differential diagnosis.

## Background

Norovirus is the major cause of epidemic viral gastroenteritis worldwide, affecting all age groups[1]. The disease is generally acute and self limited. However, there have been reported cases of chronic norovirus infections, especially in the setting of long-term immunosuppression, such as organ transplantation[2,3]. These infections can be debilitating, causing severe wasting and malnutrition, and can be confused with graft-versus-host reactions. Chronic lymphocytic leukemia (CLL) can be associated with defective immunity, leading to severe, recurrent infections[4,5]. We report two patients with CLL and hypogammaglobulinemia affected by chronic norovirus infection.

## Case Presentation

### First Case

The first patient, a 64 year old man diagnosed with CLL noted to be hypogammaglobulinemic since 2006, began having profuse, watery, non-bloody diarrhea starting in 2007, presenting initially with nausea and fever (100.5 F) which persisted for a week. The patient did not report exposure to others with diarrhea. He experienced up to 12 bowel movements daily, and was treated with intravenous immunoglobulin, octreotide, nitazoxanide, mesalamine and antimotility agents (including loperamide and diphenoxylate/atropine) without symptomatic improvement. Total parenteral nutrition (TPN) was initiated and continued for almost two years; a period of fasting while on TPN did not result in a reduction in stool frequency. An extensive gastroenterologic evaluation, including stool for leukocytes (none seen), fecal fat measurement (normal), gliadin antibody assay (negative), endoscopy, capsule endoscopy, and colonoscopy were performed; colonic biopsies demonstrated focal active colitis with an area of detached mucus and neutrophilic exudate but with preserved villi, while duodenal biopsy yielded normal mucosa. Extensive stool evaluation for non-viral pathogens (including *C. difficle, Camplyobacter *spp.*, Salmonella *spp., and *Yersinia; cryptosporidium, microsporidium *and trichrome stains; as well as *Giardia lamblia *[including antigen assay] and ova and parasite evaluation) and hormonal assays (gastrin, vasoactive intestinal polypeptide, and calcitonin) failed to yield a diagnosis. After a year of chronic diarrhea, norovirus was identified in stool by enzyme immunoassay (EIA; RIDASCREEN^® ^norovirus enzyme immunoassay; R-Biopharm, Dusseldorf, Germany)[6] in July 2008, both at our institution and at another teaching facility. His immunoglobulins at that time were profoundly low (IgG 141 mg/dl [normal: 700-1600 mg/dl]; IgA 11 mg/dl [normal: 70-400 mg/dl]; IgM 6 mg/dl [normal 40-230 mg/dl]). Stool studies for norovirus remained positive in September and December 2008, and again in February 2009. The patient expired in February 2009 from pneumonia and septic shock after receiving one cycle of rituximab, cyclophosphamide, vincristine and prednisone. As members of his family had not contracted diarrhea during his long undiagnosed course, no effort was made to isolate the patient but handwashing and attention to hygiene was advised.

### Second Case

The second patient, a 59 year old woman diagnosed with CLL in 1999 was initially found to be hypogammaglobulinemic in 2006 without prior history of recurrent infection. She had received multiple chemotherapeutic interventions over the years; her most recent treatment regimen consisted of rituximab and corticosteroids. The patient commenced intravenous immunoglobulin therapy in January 2009, following recurrent hospitalizations for pneumonia (at that time IgG 512 mg/dl, IgA < 7 mg/dl, IgM 30 mg/dl). In February 2009, after members of her immediate family experienced an undiagnosed, self-limited diarrheal illness accompanied by fever and vomiting, the patient developed bloating and daily watery, non-bloody and non-purulent diarrhea that was predominantly nocturnal (with 3-4 episodes nightly), and weight loss. Her initial presentation was associated with fever, but this abated after 10 days; she did not experience nausea or vomiting. Supportive measures (antimotility agents as noted above, active cultures, avoidance of lactose containing products, cholestyramine, and octreotide) were ineffective, and she began to require frequent infusions of intravenous fluids to combat dehydration. Infectious workup of her diarrhea revealed stool positive for the norovirus antigen (by EIA) on three occasions: March, May, and September 2009. Further gastroenterological evaluation, including endoscopy/colonoscopy, and stool evaluation for other infectious agents (culture, ova and parasite evaluation, trichrome stain, *cryptosporidium *and *microsporidium *stains, and *Giardia lamblia *antigen assay) failed to reveal another cause for her diarrhea. Duodenal biopsy in April 2009 revealed mild, active duodenitis, but no other specific diagnosis; random colonic biopsy yielded normal colonic mucosa. The patient had continuing symptoms for 11 months, had lost 10 kg despite TPN and continued to have problems with malnutrition. Diarrhea continued despite a period of fasting while on TPN. No specific treatment was offered to control the norovirus; the patient ultimately developed respiratory failure due to bilateral lobar pneumonia and died in October 2010. As her family had recovered from their diarrheal illness and had not had a recurrence during her prolonged course, no attempt to isolate the patient was made, but advice on handwashing and hygiene was given.

## Discussion

Chronic diarrhea, defined as symptoms persisting for 2 or more weeks, has a myriad of causes including infection, medication, malignancy, and inflammatory conditions. Norovirus, a calicivirus, is associated with acute gastroenteritis in humans, and is spread through the fecal-oral route, either by consumption of contaminated food or water or by direct person-person spread[1]. After an incubation period of 24 to 48 hours, patients often present with acute onset vomiting, watery non-bloody diarrhea with abdominal cramps, and nausea. Dehydration is the most common complication, especially in children and the elderly. Symptoms of this infection usually persist 24 to 60 hours, and the disease is almost always self-limited. However, hospital patient populations have been described with longer mean duration of illness, lasting 80 hours[1,3]. In the elderly, while acute symptoms abate after three to four days, other symptoms such as thirst, anorexia, lethargy, and vertigo can persist for up to 19 days, leading to delayed recovery and other complications[7,8]. The diagnosis is usually made with reverse transcriptase-polymerase chain reaction (RT-PCR) or EIA to detect the presence of norovirus in stool or emesis samples. The virus can be found in stool samples as late as 3 to 8 weeks after recovery in community based outbreaks in otherwise healthy individuals, with increasing age and children less than 2 years of age associated with long term shedding; however, no significant increase in symptoms has been found as viral concentrations diminish over time[8-11].

Gastrointestinal symptoms resulting from a variety of viral, bacterial, or parasitic pathogens, or from drug therapy or graft-versus-host disease, are common causes of morbidity and mortality in immunocompromised hosts, particularly those with solid organ transplantation requiring chronic immunosuppressant medication and patients undergoing systemic chemotherapy, or those infected with human immunodeficiency virus (see Table 1)[12]. Hypogammaglobulinemia in particular appears to be a contributing factor in recurring cytomegalovirus infection in organ transplant patients, leading to severe gastrointestinal disease[12].

**Table 1:** Causes of chronic diarrhea in patients with immunodeficiency [18,19]

Infectious Causes	Bacteria	*Escherichia coli*
		*Salmonella*
		*Shigella*
		*Campylobacter jejuni*
		*Clostridium difficile*
		*Mycoabacterium tuberculosis*
		*Mycobacterium avium *complex (MAC)
	
	Parasites	*Cryptosporidium*
		*Microsporidium*
		*Isospora Belli*
		*Giardia lamblia*
		*Entamoeba histolytica*
	
	Viral Infections	Cytomegalovirus
		Herpes virus
		HIV (AIDS enteropathy)
		Rotavirus
		Astrovirus
		Norovirus
	
	Fungus	Candida
	
	Other Causes	Invasive bacterial enteritis
Non-Infectious Causes	Treatment-related	Radiation enteritis--acute/chronic
		Graft-versus-host disease
		Chemotherapy--fluoropyrimidines, irinotecan
		Other medication side-effects
	
	Malabsorption	Small bowel bacterial overgrowth
		Lymphoma
		Kaposi sarcoma
		Pancreatic insufficiency--cytomegalovirus, MAC, medications
	
	Other Causes	Irritable bowel syndrome
		Functional disorders

More prolonged shedding and higher concentrations of norovirus have also been described in immunocompromised hosts, including congenital conditions, such as severe combined immunodeficiency and T-cell immunodeficiency, and acquired immunodeficiency, such as the setting of transplantation and patients with solid tumors receiving cytotoxic chemotherapy[1-3,12-14]. One study of pediatric oncology patients described norovirus shedding for durations exceeding 100 days--one patient had detectable norovirus detected for 420 days--leading to severe weight loss and growth retardation[13]. The case of a patient infected with human immunodeficiency virus (HIV) experiencing chronic norovirus infection and persistent, voluminous diarrhea treated with intravenous immunoglobulin was reported recently; no significant improvement was seen, although the patient was not noted to be hypogammaglobulinemic [14].

Norovirus appears to be a rare cause of chronic diarrhea, and its diagnosis is reliant on stool testing for presence of the virus, as characteristic pathological findings, simpler stool culture or routinely-applied clinical screening assays are lacking; consequently, it is conceivable that chronic norovirus-associated diarrhea may be more common than currently appreciated in patients with a variety of immunodeficiency disorders. Additionally, specific and effective therapies are not currently available for this condition. In our two cases intravenous gamma globulin did not result in amelioration of symptoms or eradication of the virus.

Patients with CLL have a number of immune system defects that evolve over time, including disordered B-cell function with decreased production of normal B-cells and abnormal production of immune globulins [15], suppressed T-cell function [16], and ultimately hypogammaglobulinemia and neutropenia in advanced cases [17]. Which defect, or combination of defects, is crucial to the development of persistent norovirus infection is unknown. In the two present cases, hypogammaglobulinemia was a common finding; the first case had not yet been treated with chemotherapy or anti CD-20 monoclonal antibody therapy at the time of development of norovirus-associated diarrhea.

## Conclusions

While CLL associated with hypogammaglobulinemia is commonly linked to recurring infection,[5] our literature search failed to find other reports of patients with CLL suffering from persistent diarrhea as a result of chronic norovirus infection. Enteric viral infections, including norovirus, should be considered in the differential diagnosis of severe, persistent diarrhea in all immunocompromised patients--including CLL--that may result in severe morbidity and mortality in those affected.

## Declaration of Competing interests

The authors declare that they have no competing interests.

## Authors' contributions

TC was responsible for assembling the cases and background information, and writing the manuscript. GMJ, RS and WCM participated in reviewing the cases, drafting and editing the manuscript, and obtaining consent for publication. GMJ conceived the study. All authors read and approved the final manuscript.

## Consent

Written informed consent was obtained from the patients' families for publication of these case reports. Copies of the written consents are available for review by the Editor-in-Chief of this journal.

## Authors' Information

TC is a fellow in hematology oncology at Baystate Regional Cancer Program. GMJ is medical director, Comprehensive Breast Center; RS is medical director of adult hematology; and WCM is medical director, Cancer Services; all at Baystate Regional Cancer Program. GMJ and WCM are associate professors of medicine, and RS assistant professor of medicine, at Tufts University School of Medicine.

## Pre-publication history

The pre-publication history for this paper can be accessed here:

http://www.biomedcentral.com/1471-2334/11/131/prepub
